# Supplementary material for: Relationship between high shear stress and OCT-verified thin-cap fibroatheroma in patients with coronary artery disease
Source: PLoS One. 2020 Dec 17;15(12):e0244015. doi: 10.1371/journal.pone.0244015 (PMC7746187; doi:10.1371/journal.pone.0244015)
Supplement: S1 Fig — OCT detected TCFA with minimal fibrous cap thickness 50 μm (inset) and a 305° lipid arc (A); large attenuated plaque detected by IVUS with maximal lipid core burden index (LCBI) within a 4 mm segment 465 detected by NIRS. (DOCX) [file pone.0244015.s001.docx]

**S1 Fig.** Representative multimodality images of a study lesion. OCT detected TCFA with minimal fibrous cap thickness 50 µm (inset) and a 305° lipid arc (A); large attenuated plaque detected by IVUS with maximal lipid core burden index (LCBI) within a 4 mm segment 465 detected by NIRS.


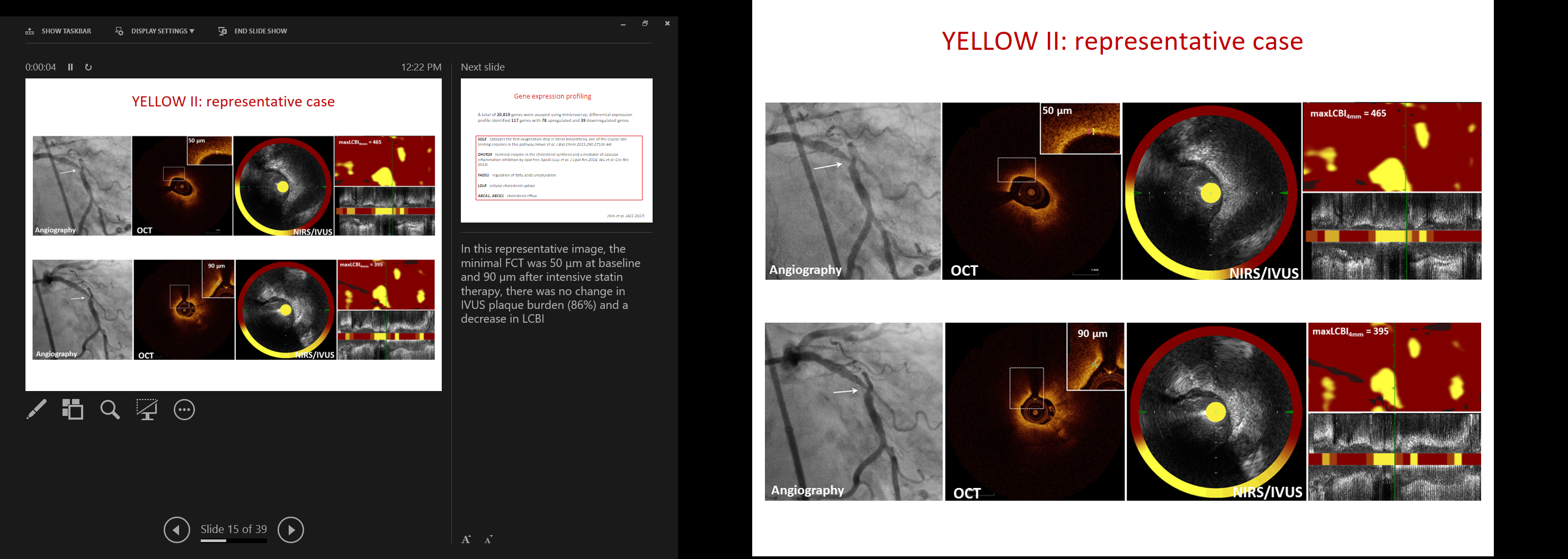


Lipid arc 305°

**C**

**D**

**B**

**A**
