# Supplementary material for: Relationship between high shear stress and OCT-verified thin-cap fibroatheroma in patients with coronary artery disease
Source: PLoS One. 2020 Dec 17;15(12):e0244015. doi: 10.1371/journal.pone.0244015 (PMC7746187; doi:10.1371/journal.pone.0244015)
Supplement: S2 Fig — (DOCX) [file pone.0244015.s002.docx]

**S2 Fig**. **Receiver-operating characteristic curve to predict thin-cap fibroatheroma lesions using angiography-based CFD analysis of wall shear stress in obstructive coronary lesions**.


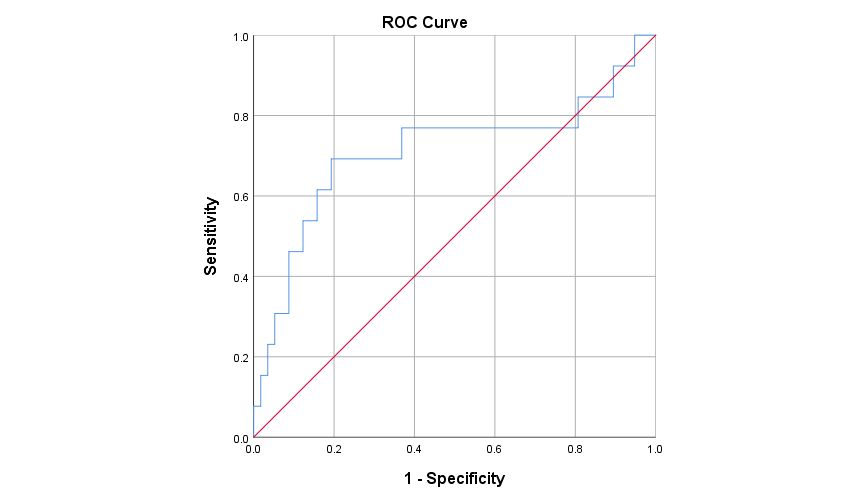


**WSS proximal**

Sensitivity = 0.77

Specificity = 0.63

**6.79 Pa**
