# Supplementary material for: Relationship between high shear stress and OCT-verified thin-cap fibroatheroma in patients with coronary artery disease
Source: PLoS One. 2020 Dec 17;15(12):e0244015. doi: 10.1371/journal.pone.0244015 (PMC7746187; doi:10.1371/journal.pone.0244015)
Supplement: S1 Table — (DOCX) [file pone.0244015.s003.docx]

S1 Table. Angiographically derived measurements and computational fluid dynamics: maximal WSS

|  | TCFA  (n=13) | No TCFA  (n=57) | P-value |
| --- | --- | --- | --- |
| Total lesion WSS (max), Pa | 65.8 (31.3, 98.7) | 44.2 (23.7, 67.6) | 0.04 |
| Upstream WSS (max), Pa | 8.5 (6.2, 21.5) | 8.7 (4.4, 21.7) | 0.63 |
| Proximal WSS (max), Pa | 34.6 (14.7, 54.5) | 13.6 (8.6, 29.6) | 0.04 |
| Middle WSS (max), Pa | 60.5 (25.1, 91.3) | 34.9 (18.9, 58.8) | 0.12 |
| Distal WSS (max), Pa | 24.6 (13.3, 48.9) | 16.4 (8.8, 34.0) | 0.25 |
| Downstream WSS (max), Pa | 14.7 (6.0, 42.7) | 13.8 (7.0, 30.1) | 0.88 |

Values are mean ± SD or median (interquartile range); TCFA= thin-cap fibroatheroma; DS= diameter stenosis; WSS= wall shear stress.
