# Supplementary material for: Relationship between high shear stress and OCT-verified thin-cap fibroatheroma in patients with coronary artery disease
Source: PLoS One. 2020 Dec 17;15(12):e0244015. doi: 10.1371/journal.pone.0244015 (PMC7746187; doi:10.1371/journal.pone.0244015)
Supplement: S2 Table — (DOCX) [file pone.0244015.s004.docx]

S2 Table. Relationship between TCFA and maximal WSS

|  | Odds ratio | 95%CI | P value |
| --- | --- | --- | --- |
| Total lesion WSS (max), Pa | 1.017 | 1.001-1.034 | 0.04 |
| Upstream WSS (max), Pa | 0.996 | 0.958-1.035 | 0.84 |
| Proximal WSS (max), Pa | 1.014 | 0.993-1.036 | 0.20 |
| Middle WSS (max), Pa | 1.013 | 0.995-1.032 | 0.16 |
| Distal WSS (max), Pa | 1.010 | 0.990-1.031 | 0.32 |
| Downstream WSS (max), Pa | 0.903 | 0.968-1.038 | 0.90 |
| Total lesion WSS (max), Pa (adjusted for 3D contrast velocity) | 1.012 | 0.988-1.035 | 0.33 |
| Proximal WSS (max), Pa (adjusted for 3D contrast velocity) | 1.015 | 0.992-1.037 | 0.83 |
